# Methylphenidate treatment of a Chinese boy with Becker muscular dystrophy combined with attention deficit hyperactivity disorder: a case report

**DOI:** 10.3389/fnins.2024.1459582

**Published:** 2024-11-26

**Authors:** Fang Shen, Hui Zhou

**Affiliations:** Department of Pediatrics, West China Second University Hospital, Sichuan University, Key Laboratory of Birth Defects and Related Diseases of Women and Children of Ministry of Education (MOE), Chengdu, Sichuan, China

**Keywords:** Becker muscular dystrophy, Duchenne muscular dystrophy, attention deficit hyperactivity disorder, methylphenidate, gene mutation

## Abstract

**Background:**

Becker muscular dystrophy (BMD) is an X-linked recessive inherited disorder characterized by slowly progressing muscle weakness of the legs and pelvis, caused by mutations in the DMD gene, which encodes dystrophin protein. Different from Duchenne Muscular Dystrophy (DMD), in which dystrophin is completely absent in muscle tissue, while in BMD, the dystrophin gene can express some protein, but not enough. It has also been shown that a proportion of patients with DMD suffer from attention deficit hyperactivity disorder (ADHD), and the use of the stimulant methylphenidate has been suggested for the treatment of patients with DMD in combination with ADHD. However, there are no case reports on the treatment of co-occurring ADHD in BMD.

**Case presentation:**

The patient was a 9-year-old boy who presented with elevated serum creatine kinase levels and inattention. The magnetic resonance imaging of the thigh muscles of both lower limbs suggested partial fatty infiltration of the gluteus maximus muscle bilaterally, and a novel heterozygous mutation (c.31 + 6 T > C) was identified in the DMD gene by Next Generation Sequencing (NGS) and the sequencing results were verified by using the Sanger method. The child was also diagnosed with co-morbid ADHD after a thorough evaluation and considering this new diagnosis, we started treatment with methylphenidate at a dose of 18 mg/day, and after 6 months of treatment, he showed a significant improvement in his attention span.

**Conclusion:**

We identified a novel heterozygous mutation in the DMD gene, which will expand the spectrum of pathogenic variants in BMD. Simultaneously, methylphenidate treatment significantly improved attention in children with BMD co-morbid with ADHD, and this study provides value for future therapeutic protocols for BMD combined with ADHD. However, to the best of our knowledge, this is the only reported case report on the treatment of BMD co-morbid ADHD. So further studies are needed to determine the interrelationship between these disorders and their treatment.

## 1 Introduction

DMD and BMD are X-linked recessive disorders caused by mutations in the DMD gene, which encodes an anti-myasthenia gravis protein. They usually develop in boys, and dystrophin proteins are expressed mainly in skeletal and cardiac muscle, where they play an important role in maintaining muscle fiber structure and function (Mah et al., 2011). Thus, missing or dysfunctional dystrophin proteins predominantly affect skeletal and cardiac muscle, and in addition, specific types of dystrophin isoforms are expressed in the brain. A meta-analysis by Salari et al. (2022) in 2022 showed that the Americas had the highest prevalence of muscular dystrophy at 5.1 per 100,000 people. In addition, the overall global prevalence of DMD and BMD was estimated to be 4.8 per 100,000 and 1.6 per 100,000, respectively. DMD is a severe form of muscular dystrophy with a high prevalence and early onset, most often at the age of 3–5 years, initially presenting with difficulty in climbing stairs, unsteady gait and recurrent falls, and then difficulty in walking at the age of 6 years, and death from cardiac and respiratory complications at the age of 20–30 years (Falzarano et al., 2015). BMD is caused by reduced or altered amounts of dystrophin proteins, usually with mild and varied clinical symptoms, the skeletal muscle weakness of BMD develops gradually, with onset around the age of about 8 years, and maintains the ability to walk until the age of 16 years and beyond, with a long life expectancy (Taglia et al., 2015).

Monaco et al. (1988) first proposed the reading frame principle, whereby the severity of clinical symptoms depends specifically on the presence or absence of a translational reading frame. They suggested that if a mutation results in a reading frame shift, a truncated and non-functional anti-myasthenic protein is produced, resulting in a severe impairment of its expression, which manifests itself as DMD, while if a deletion of the gene leaves the reading frame unchanged in a neighboring exon, the clinical symptoms are less severe and manifest as BMD. Currently, the diagnosis of DMD/BMD is mainly based on genetic testing. Although DMD/BMD cannot be completely cured, early diagnosis and timely intervention play a crucial role in slowing down the progression of the disease and improving the long-term prognosis.

DMD/BMD, in addition to progressive muscle weakness, learning difficulties, and neurocognitive, and behavioral disorders are also common in DMD patients (Hendriksen and Vles, 2008; Snow et al., 2013; Pane et al., 2012; Anderson et al., 2002), especially ADHD, with an incidence rate of approximately 32% in DMD patients (Pane et al., 2012; Anderson et al., 2002; Polanczyk et al., 2007). Learning difficulties also occur more frequently in patients with BMD with milder clinical symptoms, while behavioral and attentional problems are more common than in the general population (Young et al., 2008). Lambert et al. (2020) reported the prevalence of neurodevelopmental delays and neuropsychiatric disorders in patients with BMD, with 77% of patients exhibiting at least one neurodevelopmental symptom, including language delay (36%), attention deficit disorder (inattention and hyperactivity symptoms, 36%) and/or autistic features (11%).

ADHD is the most common neurodevelopmental disorder in childhood, with core symptoms of age-inappropriate inattention, hyperactivity, impulsivity, and negatively affecting academic, vocational, and social skills, making it important to treat children diagnosed with ADHD promptly. Clinical guidelines currently advise starting treatment of children and adolescents >6 years of age with Food and Drug Administration (FDA) approved medications (Wolraich et al., 2019). The findings of a recent review also support this recommendation (Peterson et al., 2024). Clinical guidelines for preschool children (age 4 years to the sixth birthday) advise parent training and/or classroom behavioral interventions as the first line of treatment (Wolraich et al., 2019). The medications approved by the United States FDA for the treatment of ADHD include stimulants and non-stimulants. The primary stimulant is methylphenidate, while the primary non-stimulant is atomoxetine. The effect size for stimulant treatment in ADHD is 1, whereas the effect size for non-stimulant treatment is 0.7. Methylphenidate, a first-line drug in the treatment of school-age children and adolescents with ADHD, acts by blocking the presynaptic reuptake of norepinephrine and dopamine and improves the core symptoms of hyperactivity/impulsivity and inattention in patients with ADHD (Wolraich et al., 2019; Cortese, 2020; Rubia et al., 2014). Treatment with methylphenidate has been recommended for patients with DMD combined with ADHD (Lionarons et al., 2019), but the optimal treatment for DMD combined with ADHD is unclear, and the efficacy of stimulants has not been systematically evaluated and described in this population.

To the best of our knowledge, there are no previous case reports of methylphenidate treatment in patients with BMD co-occurring with ADHD. In this article, we report a case of a boy with BMD who had a *de novo* c.31 + 6 T > C heterozygous mutation in exon 1 of the DMD gene with co-morbid ADHD, and after treating the child with methylphenidate and rehabilitating him, his attention, motor endurance, and motor balance improved compared with the previous one, and the results of our findings broaden the spectrum of causative mutations of BMD, as well as provide value for future therapeutic options for children with BMD combined with ADHD.

## 2 Case presentation

### 2.1 Early development and family history and clinical presentation

We report a 9-year-old boy who presented to the Department of Rehabilitation Medicine of West China Second Hospital of Sichuan University with elevated serum creatine kinase levels and inattention. The child was born at full term to non-consanguineous parents and had a normal pregnancy. In terms of motor development, he could walk alone with a less stable gait than his peers at 1 year and 5 months of age at 6 years of age, he could hop on one foot and walk upstairs with ease. Now at 9 years old, he can run fast, go up and down stairs, jump continuously on both feet and one foot, and walk continuously for 1 h, but he will have pain in both legs after strenuous exercise for more than 10 min, and his motor balance, co-ordination, and motor endurance lag behind those of children of the same age. However, his speech development was normal and consistent with children of the same age.

The parents claimed that the child showed poor concentration, delayed homework, often missed knowledge points, and had poor grades after entering primary school. In addition, several of the child’s teachers reported that he needed repeated reminders to concentrate in class, but despite this, he did not disrupt the classroom, was able to initiate conversations with others, and had a good relationship with his classmates. The child has no history of serious illness, and the parents deny any family history of psychiatric or genetic disorders, as well as any conditions related to attention deficit hyperactivity disorder or muscular dystrophy.

### 2.2 Physical/psychiatric/psychological examination

The child was a 9-year-old school-age child with a height of 135.2 cm (0 SD), a weight of 30 kg (0 SD), and a body mass index of 16.4 kg/m^2^ (0 SD). There was no abnormal pigmentation of the skin all over the body, and no significant abnormalities were found in hearing, vision, heart, lungs and abdominal examination. The extremities exhibit grade IV + strength (Table 1). We evaluated the motor abilities of the child using the Bruininks-Oseretsky Test of Motor Proficiency-Second Edition, which showed that his running speed and agility, body and hand coordination, balance and strength were poor and below the average for his age group. However, his fine hand control scores were at the average for his age group. Physical examination showed that he had flat feet, hypertrophy of the gastrocnemius muscles in both calves, negative Gower sign, negative Romberg test, negative heel–knee-shin test, negative rapid alternating motion test, and normal knee-jerk reflexes.

**Table 1:** Muscle strength scale.

0	Only a trace of movement is seen or felt or fasciculations are present
1	Only a trace of movement is seen or felt or fasciculations are present
2	Muscle can move only if the resistance of gravity is removed (e.g., in the horizontal plane)
3	Muscle strength is further reduced such that the joint can be moved only against gravity with the examiner’s resistance completely removed
4	Muscle strength is reduced but muscle contraction can still move joint against resistance
5	Muscle contracts normally against full resistance

*Reference: Medical Research Council. Aids to the Examination of the Peripheral Nervous System. Memorandum no. 45. London, Her Majesty’s Stationery Office, 1981.

Based on behavioral observations conducted at the clinic, the child presented as hyperactive and could only sit quietly for about 5 min. Throughout the conversation, we noticed that the child’s eyes drifted and he was inattentive, but his speech was normal. The Wechsler Intelligence Scale for Children-Fourth Edition (WISC-IV) test result was 117, which is normal. The Diagnostic and Statistical Manual of Mental Disorders, Fifth Edition (DSM-5) results met the diagnostic criteria for ADHD. Also, the Swanson, Nolan, and Pelham, Version IV-26 items (SNAP-IV-26) examination and the Weiss Functional Impairment Rating Scale-Parent Report (WFIRS-P) showed clinical functional impairment.

### 2.3 Laboratory and imaging evaluation

**Figure 1:**
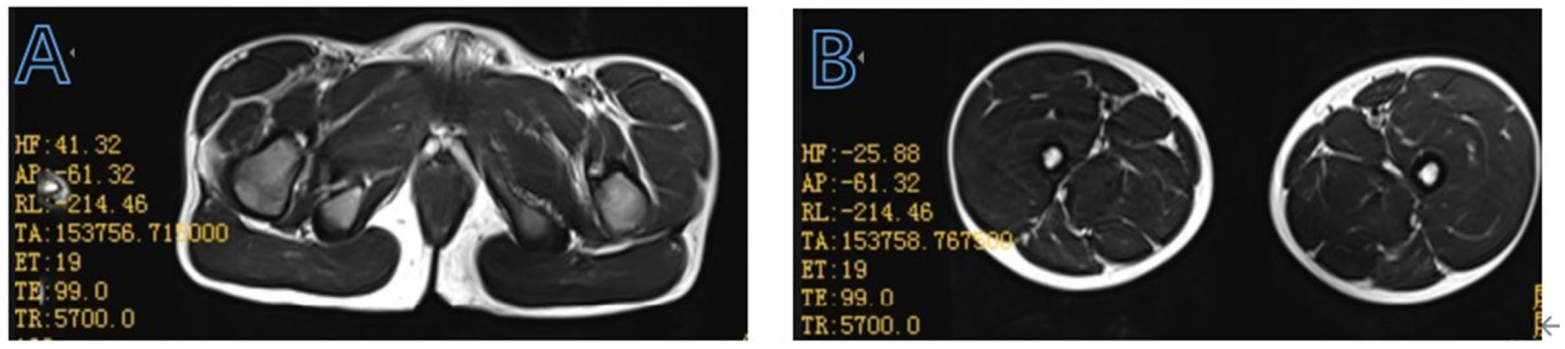
Muscle magnetic resonance T2-weighted imaging features of the enrolled patient at the pelvis (A), bilateral thigh (B).

His serum creatine kinase level was significantly elevated at 17705 U/L (normal: 39–192 U/L). Other laboratory results were as follows: troponin I 0.299 ug/L (normal: 0–0.06 ug/L), lactate dehydrogenase 650 U/L (normal: 120–246 U/L), alanine aminotransferase 160 U/L (normal: <49 U/L), aspartate aminotransferase 297 U/L (normal: <49 U/L). Blood cell counts, thyroid function, parathyroid hormone, renal function, bone density, and spinal X-ray yielded no abnormal results. The electrocardiogram showed sinus arrhythmia and rightward deviation of the electrical axis. The echocardiography demonstrated a normal heart function and structure. The magnetic resonance imaging of the thigh muscles of both lower limbs suggested partial fatty infiltration of the gluteus maximus muscle bilaterally (Figure 1), with no significant fatty replacement of his bilateral thigh muscle. Simultaneously, we communicated with the parents of the patient and recommended that the child undergo a comprehensive magnetic resonance imaging of the brain. However, the parents opted to refuse due to financial constraints and the child’s lack of cooperation, which would necessitate the use of sedatives. Therefore, the child’s clinical symptoms, physical examination, magnetic resonance imaging findings of the thigh muscles of both lower limbs, and the markedly increased creatine kinase levels together indicated to us that the boy might have BMD.

### 2.4 Genetic results

The study was approved by the ethics committee of the West China Second Hospital of Sichuan University (approval number 2024–309). In addition, we have obtained written informed consent for the release of this case from the legally authorized representative/parents of the minor subject.

**Figure 2:**
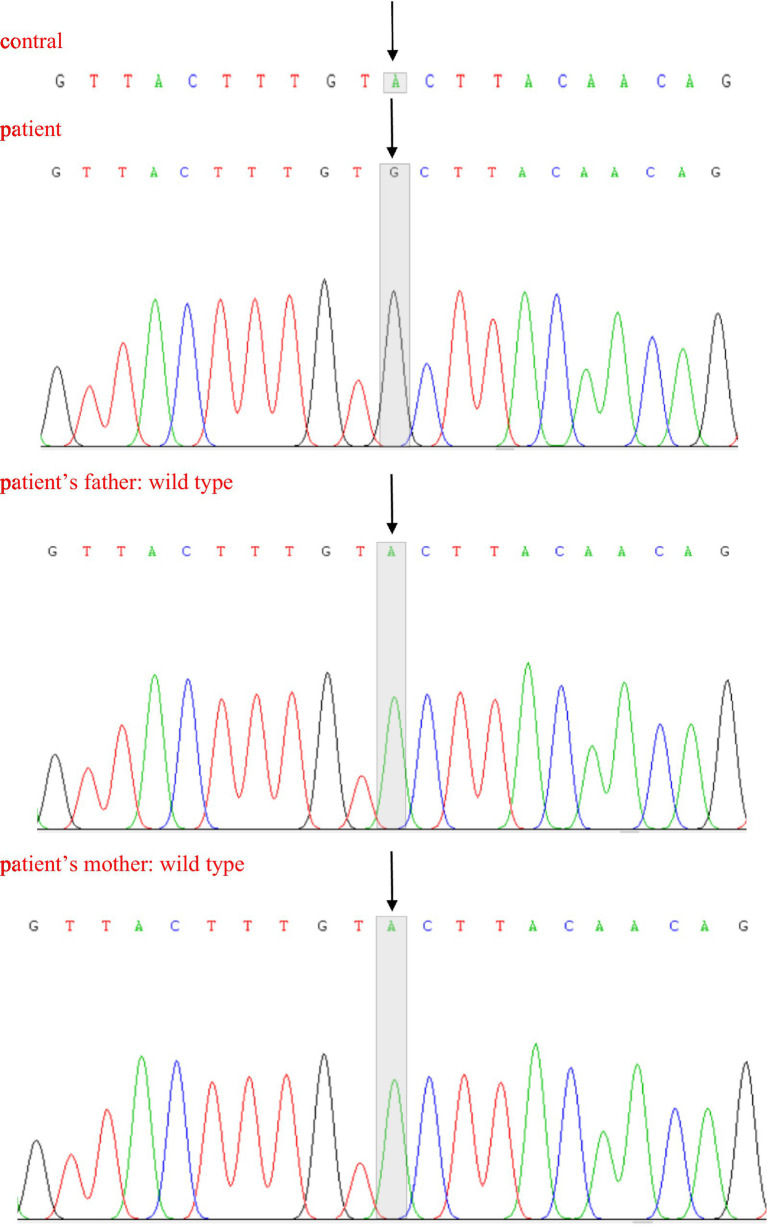
Sanger sequencing of partial DNA sequences of the DMD gene. Arrows indicate new mutation sites.

Because of the suspected BMD diagnosis of our enrolled boy, we initiated exon DNA-based genetic testing to look for DMD gene variants that contribute to the disease (Xie et al., 2023; Xie et al., 2021), including multiplex ligation-dependent probe amplification (MLPA) analysis to detect deletions or duplications in exons of the DMD gene and NGS. Blood samples (2 mL) were collected from the child and his parents for genetic analysis. MLPA analysis did not identify copy number variants (exon duplications and/or deletions) in the DMD gene, but NGS identified a clinically significant unspecified variant, which was defined according to the standards and guidelines of the American College of Medical Genetics and Genomics (ACMG). It revealed the presence of a novel heterozygous c.31 + 6 T > C mutation in exon 1 of the DMD gene (Table 2), which was shown to affect splicing using multiple protein function prediction software for gene variant-induced protein function prediction. The effect of this variant on the transcriptional function of the DMD gene could not be assessed because the parents of the child refused to have a muscle biopsy. According to our understanding, a variant at this locus (c.31 + 6 T > A) has been included in the Genome Aggregation Database (gnomAD), but this mutation in the boy in this study is a novel mutation, which has not yet been reported in the gnomAD, Online Mendelian Inheritance in Man database or ClinVar database. We used Sanger sequencing to further confirm the origin and inheritance pattern of the mutation (Figure 2), and the child was a novel heterozygous mutation, whereas both of his parents were wild-type.

**Table 2:** Mutation site details.

Gene name	Location	Gene mutation information	Americal college of medical genetics classification
DMD	ChrX: 33211276	NM_004006.2:exon1: c.31 + 6 T > C	Variant of Uncertain Significance PM6 + PM2_Supporting+PP3

PM6: this variant is a de novo mutation and has not been verified by biological relationships in family samples; PM2_Supporting: frequency in the normal population database; PP3: multiple splicing prediction software predicts that the variant affects splicing.

### 2.5 Final diagnosis, treatment and outcome

Based on the child’s clinical presentation, ancillary investigations, scale assessment and NGS results, he was diagnosed with BMD and ADHD. After the diagnosis was confirmed, the child was started to take 18 mg methylphenidate extended-release tablets orally every morning, and at the same time insisted on more than 1 h of rehabilitation (including pulling exercises and physical therapy) every day. After 6 months of methylphenidate treatment, parents and teachers used the SNAP-IV-26 test to show a significant decrease in the child’s scores on attention deficits compared to pre-treatment scores. Meanwhile, the WFIRS-P test showed a decrease in his scores in home, study and school, life skills, self-management, social activities and risk-taking activities. Thus, this result suggests that methylphenidate may significantly improve attention and social functioning in children with BMD combined with ADHD. Furthermore, after 6 months of rehabilitation, the results of the Bruininks-Oseretsky Test of Motor Proficiency-Second Edition showed an improvement in the child’s standardized scores for fine hand control, body and hand coordination, strength and agility.

## 3 Discussion

In previous studies, researchers have primarily focused on the cognitive and neurobehavioral characteristics of boys with DMD. Studies have indicated that DMD-related cognitive abnormalities, along with a marked reduction in dystrophin, the brain’s muscle protein, may lead to comprehensive alterations in neural and cortical development (Doorenweerd et al., 2017), thereby affecting intelligence, working memory, executive functions, reading and writing abilities, as well as neuropsychiatric disorders (Cotton et al., 2001; D’Angelo et al., 2011; Ricotti et al., 2016). ADHD is the most common neurobehavioral co-morbidity in DMD and has been reported in boys with BMD (Young et al., 2008). There is uncertainty regarding the cause of this, as psychological stress may also contribute to behavioral problems, although ADHD has been reported to be associated with the expression of Dp140 (mutations in exons 45–55) and Dp71 (mutations in exons 62 and 63; Felisari et al., 2000). A systematic review in 2022 (Ferrero and Rossi, 2022) highlighted the paucity of research on the cognitive aspects of BMD, while little is known about the neurodevelopmental and neuropsychiatric symptoms in boys and young men with the BMD phenotype, which may be related to its lower prevalence, later age of onset, and greater phenotypic variability than DMD. Only a small number of studies to date have demonstrated the inability of people with BMD to perform tasks requiring sustained attention, a higher co-morbid risk of learning difficulties and ADHD than in the general population, and deficits in executive functioning in people with BMD, particularly in working memory (Young et al., 2008; Biagi et al., 2021; Pezzoni et al., 2024).

ADHD is the most common neurological disorder in children and is complex in etiology and difficult to treat, with a global prevalence of up to 7.2% of children (Wolraich et al., 2019; Sayal et al., 2018). Its clinical symptoms tend to diminish with age but persist into adulthood in half of the patients (Caye et al., 2016). Children with ADHD are often associated with behavioral problems, learning disabilities, substance abuse, anxiety, depression, as well as marital and occupational problems, so early detection and intervention in children with ADHD are of great clinical and social significance. For patients after school age, disease management is mainly through medication, supplemented by behavioral interventions and psychotherapy. Methylphenidate is highly potent and works by blocking the reuptake of norepinephrine and dopamine at the synaptic cleft (Punja et al., 2016). The results of a study by Lionarons et al. using methylphenidate DMD in male patients with co-morbid ADHD showed that methylphenidate treatment significantly improved the patients’ attention without major side effects (Lionarons et al., 2019).

The boy in this study had a clear diagnosis of BMD and ADHD, and his inattention was already seriously affecting his schooling, so based on the results of the previous study, he received 18 mg methylphenidate extended-release tablets orally every morning and also adhered to rehabilitation (including pulling exercises and physical therapy) for more than 1 h every day. The purpose of rehabilitation pulling training is to maintain and improve the patient’s muscle strength, flexibility and joint range of motion, delay muscle atrophy and contracture, and improve the patient’s quality of life. The main types of pulling training include passive pulling and active pulling. Passive stretching is in which the physiotherapist and parents help the patient perform passive stretching of the major joints throughout the body daily. Active stretching is to encourage the patient to perform active stretches to the best of his/her ability, such as leg flexion and extension, and arm raising. Rehabilitation stretching exercises should be performed without causing pain. Physical therapy entails a physiotherapist designing a series of exercises, such as standing on one foot, walking a straight line, jumping, etc., to enhance the child’s coordination and balance. After 6 months of methylphenidate treatment, his parents and teachers expressed that the child’s attention had significantly improved compared to the previous period. Simultaneously, after 6 months of rehabilitation, his fine hand control, body and hand coordination, strength and agility have all improved.

This is despite the fact that methylphenidate is a first-line medication for the treatment of ADHD in children and adolescents. However, the treatment may also be affected by a range of adverse effects, such as increased blood pressure and heart rate, sleep disturbances, weight and appetite loss, restlessness, drowsiness, excoriations, tics, indifference, nausea, irritability, headaches and stomach pains (Padilha et al., 2018; Coghill et al., 2013; Dittmann et al., 2013; Coghill et al., 2014; Hennissen et al., 2017). Particularly in the DMD/BMD population, thorough medical screening to rule out contraindications and detect pre-existing symptoms (e.g., gastrointestinal problems, weight problems, chronic pain, and fatigue) prior to initiating methylphenidate therapy is critical (Latimer et al., 2017; Andrews et al., 2018; Lager and Kroksmark, 2015; Weerkamp et al., 2023).

About more than 50% of BMD patients have myocardial involvement, mostly occurring at the age of 30 years or older (Chung et al., 2016). If untreated and poorly monitored, cardiomyopathy in patients with BMD can progress to heart failure, resulting in the need for heart transplantation (Nakamura, 2015). Early cardiac treatment can delay or slow the progression of cardiomyopathy, especially in patients with BMD having extra-skeletal muscle involvement (Bourke et al., 2018). Cardiac complication in BMD correlates with life expectancy and prognosis. In the study by Nakamura et al., echocardiography showed that 40% of patients with cardiac dysfunction were under 30 years old (Nakamura et al., 2023). Therefore, cardiac assessment should be conducted from an early age. Abnormal Q waves, R/S > 1 in V 1 lead, and PR shortening on electrocardiography are characteristic of dystrophinopathies (Finsterer and Stöllberger, 2003), but <10% of patients had these findings, whereas the frequencies of axial deviation, ST, T wave, and conduction abnormalities were high and increased with age (Nakamura et al., 2023). These electrocardiography abnormalities may be a biomarker for the diagnosis of cardiomyopathy and may inform treatment.

The electrocardiogram of this patient we report also showed axis deviation. The electrocardiography before treatment with methylphenidate showed sinus arrhythmia and rightward deviation of the electrical axis. The electrocardiogram after 6 months of treatment with methylphenidate showed sinus rhythm and rightward deviation of the electrical axis. In addition, echocardiograms before and after methylphenidate treatment showed normal left ventricular ejection fraction. Meanwhile, the patient currently has no clinical symptoms of heart failure such as generalized edema, orthopnea, exertional dyspnea and easy fatigability. No signs of cardiac side effects were observed at the same time. In the future, regular reviews of electrocardiograms, echocardiograms and cardiac magnetic resonance are needed to assess cardiac involvement and provide timely treatment.

Common adverse effects in response to methylphenidate therapy include weight and appetite loss. In the case we report, before treatment with methylphenidate, the child’s weight was located at 0 SD. However, after 6 months of treatment with methylphenidate, we followed up with the child, and his parents reported that the child had occasional insomnia, but no significant loss of appetite, nausea, or gastroparesis, and his weight was still at 0 SD. Nonetheless, side effects and rebound effects may occur when methylphenidate treatment is administered, and at the individual level, good follow-up and dose management seem to be necessary. Close monitoring of growth evolution, systolic blood pressure and cardiac function may be important. In conclusion, when good side and rebound effects and therapeutic goals are achieved, methylphenidate treatment seems to be a possible solution for boys with BMD with inattention and learning problems.

## 4 Conclusion

In this study, we identified a novel heterozygous mutation in the DMD gene, which will expand the spectrum of pathogenic variants in BMD. At the same time, methylphenidate treatment significantly improves attention in children with BMD co-morbid with ADHD. Untimely diagnosis and treatment of ADHD may lead to children with adverse symptoms even in adulthood, seriously affecting their lives, learning and physical and mental health. Therefore, it is important to actively screen children with BMD for comorbidities and once the diagnosis of comorbid ADHD is confirmed, treatment should be given promptly. According to our findings, methylphenidate may be an effective drug to improve attention in children with BMD with ADHD. However, to the best of our knowledge, this is the only case report reported on the treatment of BMD combined with ADHD. So further studies are needed to determine the interrelationship between these disorders and their treatment.

## Data Availability

The original contributions presented in this study are included in this article/supplementary material, further inquiries can be directed to the corresponding author.
